# Single- and Multiple-Ascending-Dose Study of the Safety, Tolerability, and Pharmacokinetics of the Polymyxin Derivative SPR206

**DOI:** 10.1128/AAC.00739-21

**Published:** 2021-09-17

**Authors:** Jon Bruss, Troy Lister, Vipul K. Gupta, Emily Stone, Lisa Morelli, Yang Lei, David Melnick

**Affiliations:** a Spero Therapeutics Inc., Cambridge, Massachusetts, USA

**Keywords:** antimicrobial safety, pharmacokinetics, polymyxins

## Abstract

Carbapenem-resistant Acinetobacter baumannii and *Enterobacterales* are identified as urgent threats, and multidrug-resistant (MDR) Pseudomonas aeruginosa and extended-spectrum beta-lactamase (ESBL)-producing pathogens are identified as serious threats by the Centers for Disease Control and Prevention (CDC). SPR206 is a novel polymyxin derivative with potent *in vitro* and *in vivo* activity against A. baumannii, P. aeruginosa, and multiple clinically important species of *Enterobacterales*, including multidrug- and extensively drug-resistant strains. This was a first-in-human (FIH) double-blind, placebo-controlled, single-, and multiple-ascending-dose study of the safety, tolerability, and pharmacokinetics (PK) of SPR206 in 94 healthy subjects. Following intravenous (i.v.) administration (1-h infusion) at single doses of 10 mg to 400 mg and multiple doses of 25 mg to 150 mg every 8 h (q8h) for 7 days and 100 mg q8h for 14 days, SPR206 was generally safe and generally well tolerated. While the incidence of adverse events increased with dose, most were of mild severity. Systemic exposure (maximum concentration of drug in serum [*C*_max_] and area under the concentration-time curve [AUC]) to SPR206 was approximately dose proportional, time to peak concentrations ranged from 1.1 to 1.3 h, and half-life ranged from 2.4 to 4.1 h. No appreciable accumulation occurred with repeated dosing of SPR206, and trough concentrations suggest that steady state was achieved by day 2. Urinary excretion of unchanged SPR206 was dose dependent across single- (SAD) and multiple-ascending-dose (MAD) cohorts, and the percentage of dose excreted as SPR206 was up to >50%. Importantly, no evidence of nephrotoxicity was observed over 14 days of 100 mg q8h dosing of SPR206; a dosing regimen anticipated to exceed requirements for clinical efficacy. (This study has been registered at ClinicalTrials.gov under identifier NCT03792308.)

## INTRODUCTION

Among Gram-negative pathogens, antimicrobial resistance is a growing problem worldwide (1). Carbapenem-resistant Acinetobacter baumannii and *Enterobacterales* have been identified as urgent threats, and multidrug-resistant (MDR) Pseudomonas aeruginosa and extended-spectrum beta-lactamase (ESBL)-producing pathogens are considered a serious threat by the Centers for Disease Control and Prevention (CDC) and World Health Organization (WHO) (1, 2). Acinetobacter baumannii is associated with serious infections, including bacteremia, hospital-acquired and ventilator-associated bacterial pneumonia (HABP/VABP), and complicated urinary tract infections (cUTIs) (3, 4), and over 60% of infections due to A. baumannii are MDR (5–8) and are associated with excess morbidity and mortality rates of 50% or greater (3, 9–15). Carbapenem resistance in clinical isolates of P. aeruginosa approaches approximately 20% (16), and infections caused by this pathogen are associated with substantial morbidity and increased rates of mortality (17, 18). The WHO, the CDC, and others have highlighted the urgent need to identify new antimicrobial agents to treat serious infections due to MDR pathogens (1, 2, 19–21).

**FIG 1:**
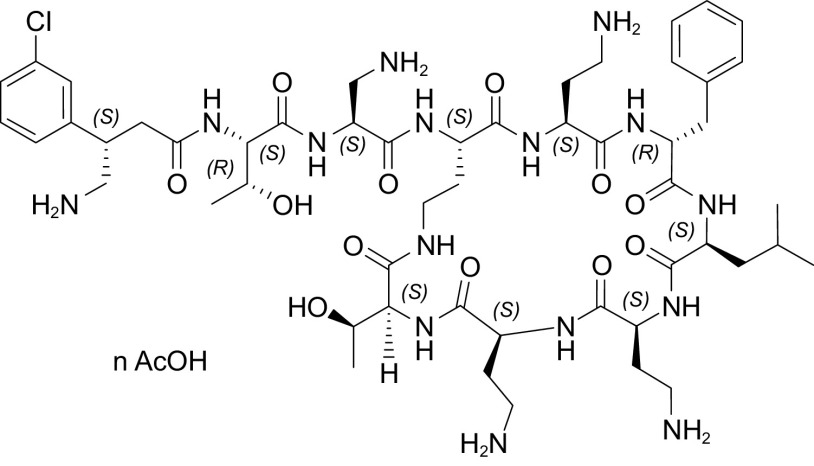
Chemical structure of SPR206.

SPR206 is a novel polymyxin derivative (Fig. 1) with potent *in vitro* and *in vivo* activity against A. baumannii, P. aeruginosa, and multiple clinically important species of *Enterobacterales*, including drug-resistant ESBL-producing and Ambler class A, B, C, and D beta-lactamase-producing strains. *In vitro* studies have shown SPR206 exhibits lower MICs (MIC_90_ range, 0.12 to 0.5 μg/ml) than colistin and meropenem against A. baumannii, Klebsiella pneumoniae, and P. aeruginosa (22–27), and *in vivo* studies in thigh, lung, and urinary tract infection models in mice indicate that SPR206 achieves efficacy endpoints (reduction in bacterial burden in CFU/g) at similar or lower required doses (mg/kg) than polymyxin B (PMB) with a change from baseline in Log_10_ of −4.6 for SPR206 and −2.8 for polymyxin B at 20 mg/kg (28–30). Nonclinical toxicology studies in mice, rats, and nonhuman primates have demonstrated that SPR206 exhibits a lower risk for kidney toxicity (nephrotoxicity) than colistin and polymyxin B, including a mouse model where no histopathological changes in the kidney were noted with SPR206 compared with all animals with polymyxin B (22, 31, 32). A suite of glycolipoprotein (GLP) repeat dose toxicology, safety pharmacology, and absorption, distribution, metabolism, and excretion (ADME) studies have shown SPR206 to be generally safe and generally well tolerated at exposures above those anticipated to be required for efficacy, with low risk for respiratory, central nervous system, or cardiovascular events and low risk for clinical drug-drug interactions. In preclinical studies, SPR206 demonstrated no apparent accumulation after repeat dosing in rats and monkeys and undergoes minimal metabolism *in vitro* and *in vivo*, and SPR206 exhibits relatively low protein binding across species, including human (<21%). SPR206 is undergoing clinical development as an intravenous (i.v.) therapy to treat serious Gram-negative infections of the lung, bloodstream, intraabdominal, and urinary tract in the hospital setting caused by MDR pathogens. This first-in-human (FIH) study evaluated the safety, tolerability, and pharmacokinetics (PK) of SPR206 in healthy subjects.

## RESULTS

### Subject disposition and baseline characteristics.

**FIG 2:**
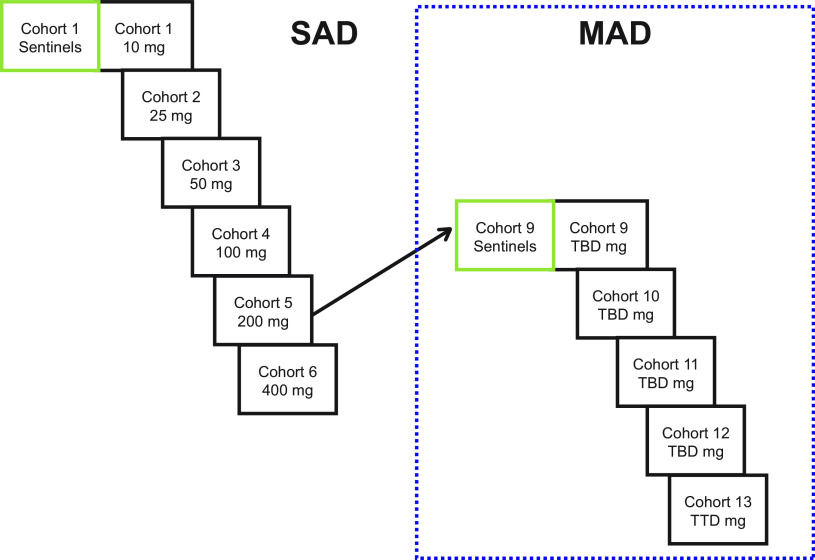
Study design.

In the single-ascending-dose (SAD) phase, 54 subjects were enrolled; 48 were randomized to one of six ascending-dose cohorts and received SPR206 at 10 mg, 25 mg, 50 mg, 100 mg, 200 mg, and 400 mg or placebo at a ratio of 3:1, respectively (Fig. 2). Due to reversible, paresthesia-like events experienced by subjects at the 400-mg dose, subjects in cohort 7 were administered a deescalated dose of 300 mg, and 6 subjects were dosed (4 SPR206 and 2 placebo) instead of 8 in other cohorts (6 SPR206 and 2 placebo). All 54 subjects completed the study and were included in the safety analysis, and all 40 subjects who received SPR206 were included in the PK analysis.

In the multiple-ascending-dose (MAD) phase, 40 subjects were randomized to one of four ascending-dose cohorts and received SPR206 at 25 mg, 50 mg, 100 mg, and 150 mg or placebo every 8 h (q8h) for 7 days at a ratio of 3 active to 1 placebo (Fig. 2). Cohort 13 (*n* = 8) received a dose of 100 mg of SPR206 or placebo q8h for 14 days at the same ratio of 3 active to 1 placebo. All 40 (100.0%) subjects completed the study and were included in the safety analysis, and all 30 subjects who received SPR206 were included in the PK analysis.

In the SAD phase, the median age for all subjects was 26.0 years (range, 18.0 to 48.0 years), mean (standard deviation) body weight was 76.4 (9.4) kg, and mean (standard deviation) body mass index (BMI) was 24.3 (2.4) kg/m^2^. The majority were white (38 [70.4%]), and 14 were Asian (25.9%). In the MAD phase, the median age was 29.5 years (range, 18.0 to 55.0 years), mean body weight was 78.4 (10.2) kg, and mean BMI was 24.9 (2.3) kg/m^2^. The majority of participants were white (31 [77.5%]), and 9 (22.5%) were Asian.

### Safety/tolerability.

**FIG 3:**
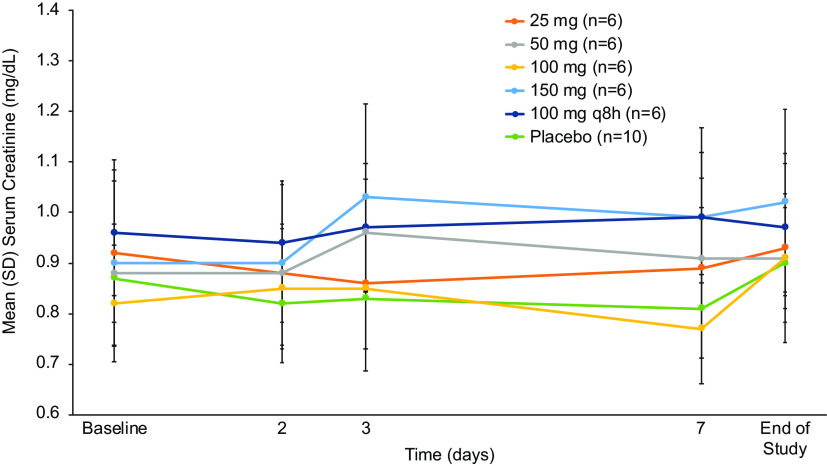
Mean (standard deviation) serum creatinine values over time with multiple ascending doses of SPR206 (safety population).

In the SAD phase, the incidence of adverse events (AEs) generally increased at doses of ≥300 mg, and in the MAD phase, the incidence of AEs was dose dependent (Table 1). Across both the SAD and MAD phases, 84% of all AEs were of mild severity, and there were no severe AEs. Two (2.1%) subjects in the highest dose group in the MAD phase (150 mg q8h) discontinued SPR206 for paresthesia and hypoesthesia. In the MAD phase, 2 subjects experienced a mild elevation of alanine aminotransferase levels >2 and <3 times the upper limit of normal (ULN) and aspartate aminotransferase levels >1.5 and <2 times the ULN, which resolved by day 15 without intervention. Alkaline phosphatase, bilirubin, and gamma-glutamyl transferase levels remained normal. One subject in the 150 mg q8h for 7 days cohort experienced mild changes in renal function: serum creatinine and calculated creatinine clearance (CrCl) remained within normal limits during dosing and follow-up, but CrCl decreased by 27% from baseline on day 7 and serum creatinine increased by 0.36 mg/dl on day 6. Of note, this subject experienced approximately 23% higher plasma area under the concentration-time curve from 0 to 8 h (AUC_0–8_) and maximum concentration of drug in serum (*C*_max_) on day 1 and day 7 relative to the mean values for the cohort. No other subjects at any dose or duration of dose experienced changes in serum creatinine (Fig. 3) or calculated creatinine clearance and no clinically significant changes in fractional excretion of calcium and magnesium or urine cation/Cr ratios (Ca and Mg) to suggest a change in renal function was observed. No serious AEs were reported, and no clinically significant changes were observed in vital signs, physical examination, or electrocardiogram (ECG) parameters.

**TABLE 1:** Incidence of treatment emergent adverse events (safety population)

Phase and adverse event parameter	No. (%) of subjects according to SPR206 group							
Single ascending dose group (no. of subjects)	10 mg (*n* = 6)	25 mg (*n* = 6)	50 mg (*n* = 6)	100 mg (*n* = 6)	200 mg (*n* = 6)	400 mg (*n* = 6)	300 mg (*n* = 4)	Pooled placebo (*n* = 14)
At least one treatment-emergent adverse event	1 (16.7)	0	1 (16.7)	1 (16.7)	2 (33.3)	6 (100)	4 (100)	4 (28.6)
At least one treatment-related adverse event	1 (16.7)	0	1 (16.7)	1 (16.7)	2 (33.3)	6 (100)	4 (100)	1 (7.1)
TEAE^*a*^ occurring in >1 subject								
Dizziness	0	0	0	0	0	5 (83.3)	4 (100)	1 (7.1)
Headache	0	0	0	0	0	3 (50.0)	1 (25.0)	0
Diarrhea	0	0	0	0	0	2 (33.3)	0	1 (7.1)
Fatigue	0	0	0	0	0	2 (33.3)	0	0
Vision blurred	0	0	0	0	0	4 (66.7)	0	0
Paresthesia	0	0	0	0	0	1 (16.7)	3 (75.0)	0
Paresthesia oral	0	0	0	0	0	0	3 (75.0)	0
Multiple ascending dose group (SPR206 doses every 8 h)	25 mg (*n* = 6) × 7 days	50 mg (*n* = 6) × 7 days	100 mg (*n* = 6) × 7 days	150 mg (*n* = 6) × 7 days	100 mg (*n* = 6) × 14 days			Pooled placebo (*n* = 10)
At least one treatment-emergent adverse event	4 (66.7)	5 (83.3)	6 (100)	6 (100)	4 (66.7)			4 (40.0)
At least one treatment-related adverse event	1 (16.7)	4 (66.7)	5 (83.3)	4 (66.7)	4 (66.7)			2 (20.0)
TEAE leading to drug withdrawal	0	0	0	2 (33.3)	0			0
TEAE occurring in >1 subject								
Dizziness	0	1 (6.7)	0	0	2 (33.3)			2 (20.0)
Headache	1 (6.7)	2 (33.3)	2 (33.3)	0	1 (16.7)			1 (10.0)
Paresthesia oral	0	2 (33.3)	1 (16.7)	2 (33.3)	1 (16.7)			1 (10.0)
Hypoesthesia oral	0	0	3 (50.0)	3 (50.0)	0			0
Constipation	0	3 (50.0)	0	1 (16.7)	0			0
Dry mouth	0	0	0	2 (33.3)	0			1 (10.0)
Infusion site phlebitis	0	1 (16.7)	1 (16.7)	2 (33.3)	1 (16.7)			0

a TEAE, treatment emergent adverse event.

### Pharmacokinetics. (i) SAD phase.

**FIG 4:**
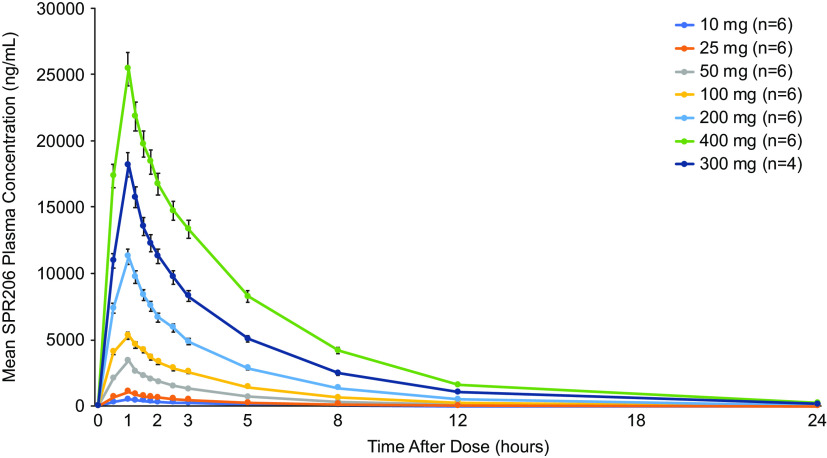
Mean (standard deviation) plasma SPR206 concentrations after single ascending doses (PK population).

Plasma SPR206 concentrations increased with increasing dose (Fig. 4). Overall, mean SPR206 concentration-time profiles showed an initial peak at the end of infusion (i.e., 1 h following the start of infusion), followed by a biexponential decline. Mean peak plasma concentrations (*C*_max_) and systemic exposure (AUC) generally increased in a dose-proportional manner with SPR206 dose (Table 2). Interindividual variability in systemic exposure to SPR206 across doses was low with geometric coefficient of variation (CV) for AUC_0–last_, AUC_0-inf_, and *C*_max_ ranging from 7.0% to 18.2%, 6.9% to 17.4%, and 6.9% to 13.8%, respectively. Dose proportionality estimates (90% confidence interval [CI]) for *C*_max_, AUC_0–last_, and AUC_0–inf_ were 1.05 (1.02, 1.08), 1.14 (1.11, 1.18), and 1.11 (1.07, 1.14), respectively. Although 90% CIs did not include the value of 1, the upper limit of all CIs was <1.2, indicating that SPR206 exposure was generally dose proportional, although strict dose proportionality cannot be concluded.

**TABLE 2:** Arithmetic mean PK parameters for SPR206 after single ascending doses (PK population)

Parameter	SPR206 (mean ± SD) according to dose						
	10 mg (*n* = 6)	25 mg (*n* = 6)	50 mg (*n* = 6)	100 mg (*n* = 6)	200 mg (*n* = 6)	400 mg (*n* = 6)	300 mg (*n* = 4)
*C*_max_ (ng/ml)	495 ± 46.5	1,286 ± 184	3,422 ± 478	5,330 ± 634	11,265 ± 1,068	25,400 ± 1,747	18,175 ± 1,733
*T*_max_ (h)^*a*^	1 (1.0–1.1)	1 (1.0–1.0)	1 (1.0–1.0)	1.1 (1.1–1.1)	1.1 (1.1–1.1)	1.1 (1.1–1.1)	1.1 (1.1–1.1)
AUC_0–8_ (h · ng/ml)	1,562 ± 203	3,806 ± 708	9,375 ± 1,098	17,324 ± 2,343	34,475 ± 2,279	89,257 ± 5978	57,733 ± 3,754
AUC_0–inf_ (h · ng/ml)	1,809 ± 258	4,443 ± 841	10,674 ± 1,223	20,402 ± 3,200	40,871 ± 3,397	10,9757 ± 7,398	71,008 ± 5,201
Half-life (h)	2.6 ± 0.1	2.8 ± 0.2	2.6 ± 0.3	3.0 ± 0.3	3.4 ± 0.7	3.7 ± 0.5	4.1 ± 0.4
CL (liters/h)	5.6 ± 0.8	5.8 ± 0.9	4.7 ± 0.5	5.0 ± 0.9	4.9 ± 0.4	3.7 ± 0.3	4.2 ± 0.3
*V*_z_ (liters)	21.0 ± 3.4	23.5 ± 4.3	17.8 ± 2.9	21.4 ± 2.8	24.0 ± 3.1	19.4 ± 3.2	25.4 ± 3.3

a Median (range).

**FIG 5:**
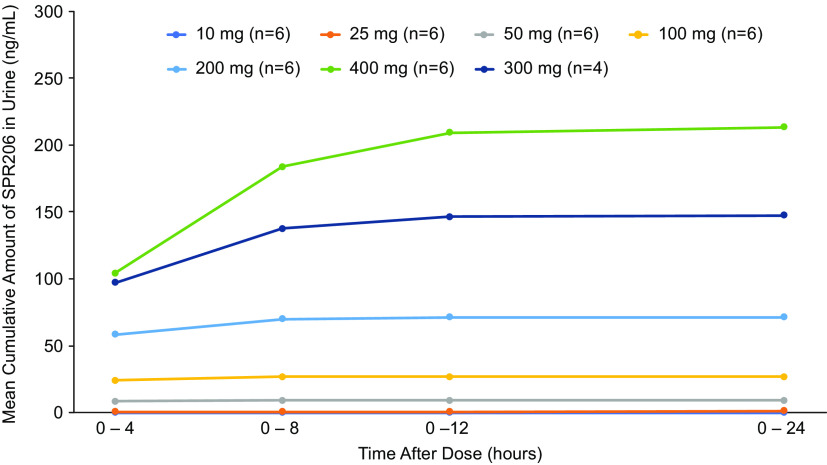
Mean cumulative amount of SPR206 excreted in urine after single ascending doses (PK population).

Mean cumulative amount of SPR206 excreted in urine increased with dose following single dose administration (Fig. 5). Generally, SPR206 was mostly excreted during the 0- to 4-h interval following the start of infusion and was low or below quantitation limit (BLQ) for the 4- to 8-h, 8- to 12-h, and 12- to 24-h collection times. Mean (standard deviation [SD]) total amount of SPR206 excreted from 0 to 24 h following the start of infusion on day 1 ranged from 0.08 mg at 10 mg up to 213.4 (24.8) mg at 400 mg (Table 3). Mean percentage excreted of SPR206 ranged from 0.7% to 53.4% after single doses of 10 to 400 mg. Renal clearance increased in a dose-proportional manner.

**TABLE 3:** SPR206 urine PK parameters in single- and multiple-dose cohorts (PK population)

SPR206 dose group	Mean ± SD^*a*^			
	Ae (mg)	CL_R_ (L/h)	Fe	Fe (%)
SAD				
10 mg (*n* = 1)	0.75	0.034	0.007	0.70
25 mg (*n* = 5)	1.33 ± 1.09	0.32 ± 0.29	0.05 ± 0.04	5.32 ± 4.38
50 mg (*n* = 6)	9.01 ± 0.95	0.85 ± 0.10	0.18 ± 0.02	18.02 ± 1.93
100 mg (*n* = 6)	26.88 ± 5.06	1.35 ± 0.34	0.27 ± 0.05	26.92 ± 5.06
200 mg (*n* = 6)	71.36 ± 7.68	1.77 ± 0.20	0.36 ± 0.04	35.68 ± 3.85
400 mg (*n* = 6)	213.42 ± 24.78	1.97 ± 0.22	0.53 ± 0.06	53.35 ± 6.21
300 mg (*n* = 4)	147.54 ± 22.49	2.13 ± 0.43	0.49 ± 0.08	49.15 ± 7.50
MAD day 1				
25 mg q8h (*n* = 5)	1.04 ± 0.67	0.24 ± 0.15	0.04 ± 0.03	4.14 ± 2.70
50 mg q8h (*n* = 6)	6.51 ± 1.83	0.82 ± 0.25	0.13 ± 0.04	13.03 ± 3.66
100 mg q8h (*n* = 6)	25.28 ± 4.86	1.55 ± 0.38	0.25 ± 0.05	25.28 ± 4.87
150 mg q8h (*n* = 6)	57.66 ± 7.86	2.13 ± 0.44	0.38 ± 0.05	38.42 ± 5.23
100 mg q8h (14 d) (*n* = 6)	24.97 ± 8.08	1.58 ± 0.39	0.25 ± 0.08	24.95 ± 8.08
MAD day 7				
25 mg q8h (*n* = 6)	1.76 ± 0.73	0.22 ± 0.06	0.07 ± 0.03	7.03 ± 2.94
50 mg q8h (*n* = 6)	8.51 ± 2.82	0.69 ± 0.20	0.17 ± 0.06	17.03 ± 5.64
100 mg q8h (*n* = 6)	33.09 ± 13.40	1.25 ± 0.50	0.33 ± 0.13	33.08 ± 13.94
150 mg q8h (*n* = 4)	81.17 ± 3.81	1.55 ± 0.17	0.54 ± 0.03	54.10 ± 1.52
100 mg q8h (14 d) (*n* = 6)	36.30 ± 6.72	1.29 ± 0.24	0.36 ± 0.07	36.30 ± 6.73

a Ae, cumulative amount of drug excreted in successive urine intervals with quantifiable urine concentrations; CL_R_, renal clearance; Fe, fraction of cumulative fraction of dose recovered in urine as unchanged drug in successive urine intervals with quantifiable urine concentrations; Fe (%), percentage fraction of cumulative fraction of dose recovered in urine as unchanged drug in successive urine intervals with quantifiable urine concentrations.

**TABLE 4:** Arithmetic mean for PK parameters for SPR206 after multiple ascending doses on day 1 and day 7 or day 14 (PK population)

Day and parameter	SPR206 (mean ± SD)				
Day 1	25 mg q8h × 7 days (*n* = 6)	50 mg q8h × 7 days (*n* = 6)	100 mg q8h × 7 days (*n* = 6)	150 mg q8h × 7 days (*n* = 6)	100 mg q8h × 14 days (*n* = 6)
*C*_max_ (ng/ml)	1,410 ± 241	2,525 ± 413	5,433 ± 678	8,990 ± 1,056	5,065 ± 1,003
*T*_max_ (h)^*a*^	1.1 (1.0, 1.3)	1.1 (1.1, 1.3)	1.1 (1.0, 1.1)	1.1 (1.0, 1.1)	1.1 (1.0, 1.3)
AUC_0–8_ (h · ng/ml)	4,391 ± 740	7,955 ± 951	16,544 ± 2,337	27,492 ± 3,452	15,573 ± 1,765
AUC_0–inf_ (h · ng/ml)	5,197 ± 945	9,243 ± 1,417	19,171 ± 2,874	30,981 ± 4,189	18,184 ± 1,244
Half-life (h)	2.7 ± 0.2	2.7 ± 0.3	2.7 ± 0.2	2.4 ± 0.2	2.5 ± 0.2
CL (liters/h)	4.9 ± 0.9	5.5 ± 0.8	5.3 ± 0.8	4.9 ± 0.6	5.5 ± 0.4
*V*_z_ (liters)	18.9 ± 2.6	21.5 ± 1.8	20.8 ± 2.9	17.1 ± 2.1	19.9 ± 3.0
Day 7 or day 14	25 mg q8h × 7 days (*n* = 6)	50 mg q8h × 7 days (*n* = 6)	100 mg q8h × 7 days (*n* = 6)	150 mg q8h × 7 days (*n* = 6)	100 mg q8h × 14 days (*n* = 6)
*C*_max_ (ng/ml)	1,607 ± 300	2,897 ± 270	6,345 ± 1099	11,518 ± 1,901	6,423 ± 841
*T*_max_ (h)^*a*^	1.1 (1.0, 1.3)	1.1 (1.0, 1.2)	1.1 (1.0, 1.1)	1.1 (1.1, 1.1)	1.1 (1.0, 1.1)
AUC_0–8_ (h · ng/ml)	5,881 ± 1,166	10,050 ± 1,050	21,340 ± 2,857	41,475 ± 6,083	22,277 ± 2,574
AUC_0–48_ (h · ng/ml)	7,681 ± 1,726	12,435 ± 1,796	27,531 ± 4,610	55,505 ± 7,838	29,828 ± 5,112
AUC_0–inf_ (h · ng/ml)	7,690 ± 1,732	12,443 ± 1,802	27,671 ± 4,762	56,007 ± 7,874	30,412 ± 5,699
AUC_%ext_ (h · ng/ml)	10.0 ± 4.5	6.9 ± 2.5	1.9 ± 0.4	0.9 ± 0.1	3.3 ± 1.4
Half-life (h)	4.0 ± 0.6	3.5 ± 0.9	5.3 ± 1.4	5.9 ± 0.3	9.6 ± 8.1
CL (liters/h)	4.4 ± 0.9	5.0 ± 0.5	4.8 ± 0.6	3.7 ± 0.5	4.5 ± 0.5
*V*_z_ (liters)	21.3 ± 3.6	21.5 ± 2.2	22.5 ± 3.5	19.6 ± 3.2	23.9 ± 4.0

a Median (range).

### (ii) MAD phase.

**FIG 6:**
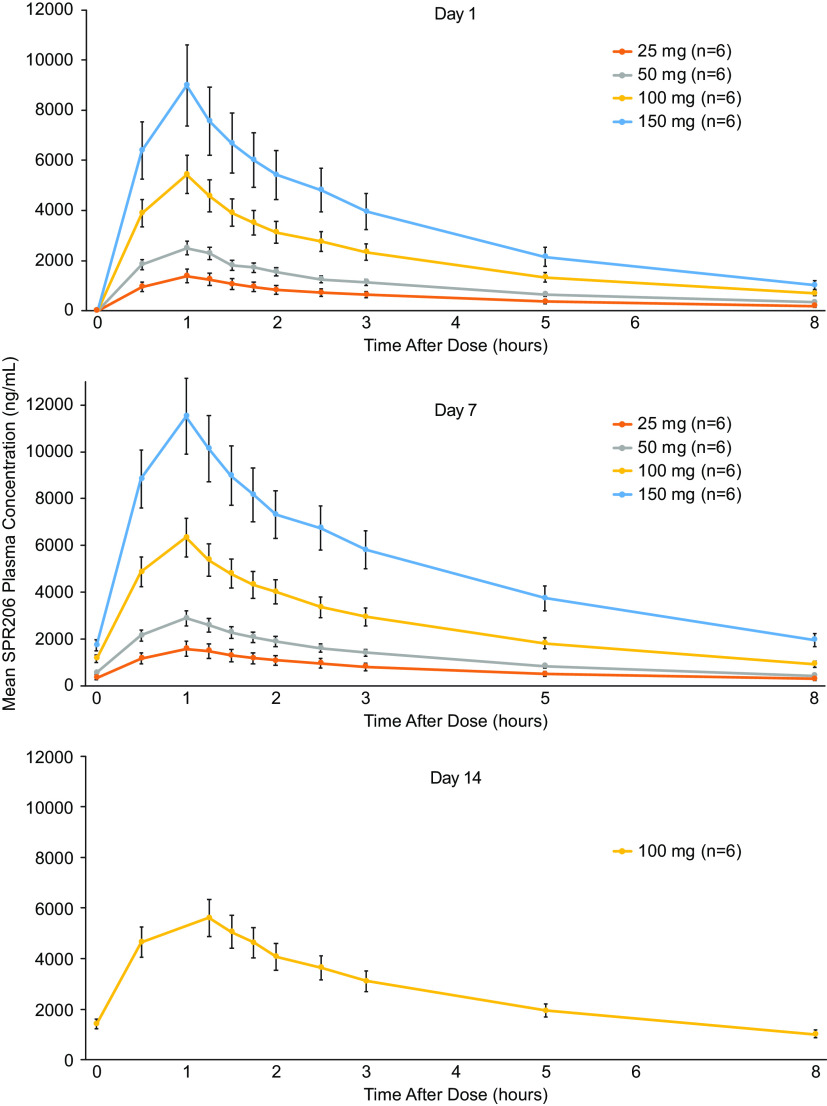
Mean (standard deviation) plasma SPR206 plasma concentrations following multiple ascending doses on day 1, day 7, and day 14 (PK population).

**FIG 7:**
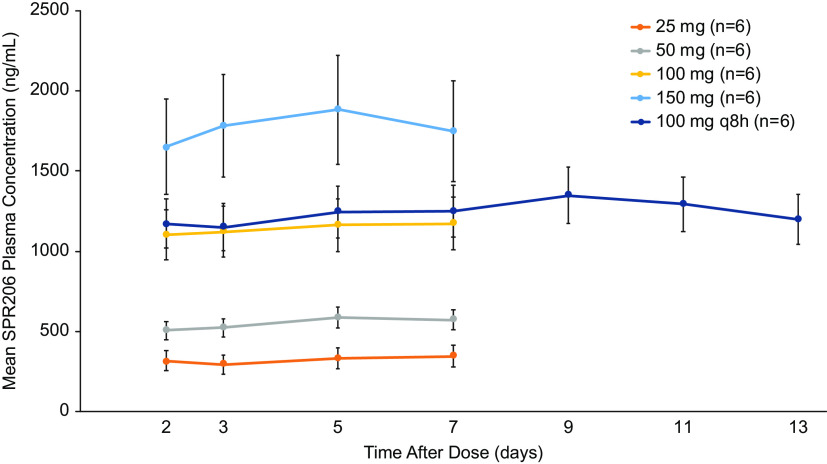
Mean (standard deviation) SPR206 trough concentrations following 7-day and 14-day administration.

For each cohort, mean plasma SPR206 levels increased with increasing dose levels following q8h dosing for 7 or 14 days (Fig. 6). Systemic exposure (AUC) to SPR206 increased in a dose-proportional manner (Table 4). Time to maximum concentration of drug in serum (*T*_max_) was 1.1 h across all dose regimens. SPR206 steady state was achieved by day 2 with q8h dosing based on *C*_trough_ levels. Similar to SAD, interindividual variability in systemic exposure to SPR206 was generally low with geometric CVs for AUC_0–last_, AUC_0–inf_, and *C*_max_ ranging from 11.8% to 16.8%, 6.8% to 18.7%, and 10.9% to 21.7% on day 1 and 13.8% to 24.3%, 13.7% to 24.6%, and 9.5% to 17.7% on day 7, respectively, across all doses. Based on estimates of the exponent from the power model, estimates (90% CI) for *C*_max_, AUC_0–last_, and AUC_0–inf_ were 1.03 (0.96, 1.10), 1.07 (0.98, 1.16), and 1.03 (0.94, 1.12), respectively, and for *C*_max_, AUC_0–last_, and AUC_0–inf_, the 90% CIs included the value of 1 for all three parameters. Trough concentrations of SPR206 remained constant during the 7- and 14-day dosing periods (Fig. 7).

For cohorts 9 through 12 (q8h dosing for 7 days), the mean (SD) total amount of SPR206 excreted in urine on day 7 (0 to 24 h) following the start of infusion increased with increasing dose ranging from 1.8 mg (0.7) for the 25-mg dose up to 81.2 mg (3.8) for the 150-mg dose. For cohort 13 (100 mg q8h dosing for 14 days), the mean total amount of SPR206 excreted in urine on day 14 was 36.3 mg (6.7). Similar to the SAD phase, the majority of SPR206 excreted in urine was observed during the 0- to 4-h collection period on day 7 or day 14 (cohort 13), and the amount excreted generally decreased for each subsequent collection interval. The mean percentage of dose excreted in urine increased with increasing dose level (Table 3). On day 1, mean (SD) percentage dose excreted was 4.1 (2.7), 13.0 (3.7), 25.3 (4.9), 25.0 (8.1), and 38.4 (5.2) with 25-mg, 50-mg, 100-mg (7 days), 100-mg (14 days), and 150-mg doses, respectively. On day 7, mean (SD) percentage excreted was 7.0 (2.9), 17.0 (5.6), 33.1 (13.4), 36.3 (6.7), and 54.1 (2.5) with 25-mg, 50-mg, 100-mg (7 days), 100-mg (14 days), and 150-mg doses, respectively. Renal clearance increased in a dose-proportional manner. No clinically significant changes in urine cation/Cr ratios (Ca and Mg) were observed.

## DISCUSSION

Results from this FIH study demonstrated that SPR206 was generally well tolerated after single and multiple doses with no serious AEs. The most common AEs were mild paresthesia, dizziness, and headache with a greater frequency at higher doses. Five paresthesia events considered nervous system disorders occurred in 4 (7.4%) subjects at 300- or 400-mg doses in the SAD cohort. Eight events of oral hypoesthesia in 6 subjects and 6 events of oral paresthesia occurred in 5 subjects in the MAD portion. All paresthesia events were of mild severity, and none led to study discontinuation. Mild, reversible paresthesia-like events are well described with the polymyxin class of antibiotics (33). In this study, there was only 1 report of mild, self-resolved paresthesia over 14 days of dosing at 100 mg every 8 h. SPR206 had no clinically significant effect on renal function as assessed from serum creatinine and creatinine clearance at doses up to 100 mg administered every 8 h for 14 days. Although remaining well within normal limits, slight changes in a single subject in serum creatinine and calculated creatinine clearance on the last day of dosing (day 7) in the highest dose group of 150 mg q8h may indicate a mild decline in renal function caused by SPR206. Further study in critically ill patients with severe infections due to resistant bacteria are needed to fully assess the impact of SPR206 on renal function. In addition, SPR206 had no effect on the ECG and no clinically significant effect on liver function.

SPR206 exhibited a dose-proportional PK profile over the range of doses studied in both SAD and MAD phases. Minimal accumulation of SPR206 occurred during the MAD phase as evidenced by low accumulation ratios for *C*_max_ (1.1 to 1.3) and AUC_0–inf_ (1.3 to 1.8). Intersubject variability in exposure was low, ranging from 10% to 25% across dose cohorts. Half-life ranged from 2.4 to 4.1 h, which supports q8h dosing. Trough concentrations (*C*_trough_) of SPR206 indicated that the steady state was achieved by day 2 with repeat dosing. Urinary excretion of unchanged SPR206 was dose dependent across single- and multiple-ascending-dose cohorts, and approximately 50% of the dose was excreted as SPR206. These data will be useful for determining dose and regimen for future studies of SPR206.

Aminoglycosides and polymyxins often are reserved for treating patients with serious infections due to MDR Gram-negative bacteria; however, a major limitation of these drugs is the increased risk for renal toxicity (34–36). The use of aminoglycosides requires regular therapeutic drug monitoring to assess drug concentrations and to adjust dosages as necessary to maintain plasma concentrations within a narrow therapeutic window and to avoid toxicity (37, 38). SPR206 is a novel polymyxin B (PMB) analogue with a β-branched aminobutyrate N terminus, bearing an aryl substituent that has been shown to possess lower kidney cell cytotoxicity and lower exposure in the kidney of rats than PMB (31, 32). The discovery of SPR206 was the culmination of an extensive medicinal chemistry effort focused on significantly improving the nephrotoxicity profile compared to current polymyxins (31), while retaining potent *in vitro* and *in vivo* activity against MDR Gram-negative pathogens. The first-in-human phase 1 study of SPR206 presented here demonstrates SPR206 to be devoid of nephrotoxicity at doses up to and including 100 mg q8h for 14 days in healthy subjects, whereas it is documented that colistin use at the recommended dosing regimen is associated with up to 60% incidence of acute kidney injury (38). As such, SPR206 has the potential to offer a spectrum of activity similar or superior to current polymyxins against MDR pathogens causing serious infections but with a meaningful improvement in safety profile and clinical outcome.

SPR206 is being developed for i.v. administration in the hospital settings to treat serious infections of the lung, blood, and urinary tract caused by resistant Gram-negative pathogens. SPR206 may offer an alternative to polymyxin- and aminoglycoside-based therapy in hospitalized patients with serious infections.

## MATERIALS AND METHODS

This study was conducted according to the principles of the Declaration of Helsinki and Guidance on Good Clinical Practice. The study protocol, amendments, and informed consent forms were reviewed and approved by an independent ethics committee. All subjects provided written informed consent prior to participating in any study activities. This study was registered at ClinicalTrials.gov under identifier NCT03792308.

### Study design.

This was a single-center, phase 1, randomized, double-blind, placebo-controlled, first-in-human study to assess the safety, tolerability, and PK of SPR206 following SAD and MAD administrations (Fig. 7). In the SAD phase, subjects received a single i.v. dose of SPR206, and in the MAD phase, subjects received i.v. doses of SPR206 every 8 h (q8h) over a period of 7 to 14 days. SPR206 was administered as a 1 h i.v. infusion.

In the SAD phase, healthy subjects were screened within 28 days prior to dosing and admitted to the clinical facility on day −1. A single i.v. dose of SPR206 or placebo was administered on day 1. Following completion of all safety assessments and sampling for PK analyses, subjects were discharged on day 2. A follow-up visit occurred 5 to 7 days after day 1 dosing.

In the SAD phase, subjects were randomized to one of seven cohorts consisting of 10-, 25-, 50-, 100-, 200-, 300-, and 400-mg doses of SPR206. Within each cohort, two subjects received placebo and six subjects received SPR206. Two subjects (sentinels) were dosed with SPR206 or placebo 48 h prior to the remaining subjects. The remaining six subjects were only dosed after no safety concerns were identified in the sentinel subjects. After each dose cohort had completed study drug dosing and safety evaluations, a safety monitoring group (SMG) reviewed blinded cumulative safety data (including day 5 to 7 follow-up data) to confirm the safety and tolerability of SPR206. Blood and urine samples were collected for assessment of PK parameters.

In the MAD phase, all subjects were admitted to the clinical facility on day −1. Dosing commenced on the morning of day 1. Three doses were administered daily at approximately 8 (±0.5)-hour intervals for a total of 7 consecutive days (cohorts 9 through 12) and 14 consecutive days (cohort 13). The last dose was administered on the morning of day 14. Subjects were discharged on day 16 following completion of all PK sample collection and safety assessments, and a follow-up visit occurred 12 to 14 days after the last dose.

The MAD phase began after completion of the SAD phase, and the appropriate starting dose level was established. Two subjects received placebo, and six subjects received SPR206 doses of 25, 50, 100, and 150 mg q8h for 7 days and 100 mg q8h for 14 days. In each cohort of the MAD, two subjects (sentinels) began dosing with SPR206 or placebo 72 h prior to the remaining subjects in this cohort. The remaining six subjects were only dosed after no safety concerns were identified by the principal investigator. After each MAD dose cohort had completed administration of study drug and all evaluations, the safety monitoring group reviewed blinded cumulative safety data (including the day 19 to 21 follow-up data) to confirm the safety and tolerability of the study drug. Blood and urine samples were collected to measure SPR206 drug levels to determine SPR206 PK parameters.

### Subject selection.

Healthy adult subjects aged 18 to 55 years with a body mass index (BMI) of 18.5 to 29.9 kg/m^2^ inclusive and weight between 55 and 100 kg were eligible. Subjects were medically healthy with no clinically significant abnormalities based on physical examination, vital signs, ECG, and clinical laboratory testing. Subjects were excluded for any clinically significant medical condition or laboratory abnormality; presence or history of any clinically significant cardiac abnormalities including clinically significant ECG abnormalities; history of seizure disorders; history of Clostridium difficile infection; positive human immunodeficiency virus (HIV) antibody, hepatitis B surface antigen (HBsAg), or hepatitis C antibody; positive urine drug/alcohol test or history of substance or alcohol abuse; documented hypersensitivity or anaphylaxis to any medication; use of tobacco or nicotine-containing products within 30 days; receipt of any investigational drug or participation in a clinical trial within 30 days; or use of any prescription or over-the-counter medications within 7 days of randomization.

### Study assessments.

Safety assessments included clinical laboratory testing (hematology, coagulation, serum chemistry, urinalysis), vital signs (blood pressure, heart rate, body temperature, respiratory rate), physical examination, and triplicate 12-lead ECG to assess corrected QT interval by Fredericia (QTcF). In the SAD phase, continuous cardiac monitoring was performed from 1 h predose through 24 h postdose. Adverse events were recorded at each study visit.

In the MAD phase, 24-h creatinine clearance (CrCl) based on plasma and urine creatinine concentrations was determined prior to any dosing and following the last dose on day 14. Serum creatinine concentrations were measured from the clinical laboratory tests performed on days −1 and 15. Urine creatinine concentration were measured using 24 h collections prior to the first dose on day 1 and over 24 to 48 h following the start of infusion of the last dose (day 14).

### Pharmacokinetic analysis.

Maximum plasma concentration (*C*_max_), area under the concentration-time curve from time zero to last measurable time point (AUC_0–t_), area under the concentration-time curve from time zero to infinity (AUC_0–inf_), area under the concentration-time curve from time zero to tau (AUC_0–tau_), time to maximum concentration (*T*_max_), terminal elimination rate constant (*k*_el_), terminal half-life (*t*_1/2_), terminal clearance (CL), and volume of distribution (*V*_z_) were determined. In addition, area under the concentration-time curve from 0 to 24 h after the start of infusion (AUC_0–24_) was determined for the SAD phase, and area under the concentration-time curve from time zero to 8 h from start of infusion (AUC_0–8_) on day 1 and 0 to 48 h (AUC_0–48_) following the last dose on day 14 as well as predose trough concentrations were determined for the MAD phase. Urine parameters included cumulative amount of drug excreted in successive urine intervals (Ae), renal clearance (CL_R_), fraction of cumulative fraction of dose recovered in urine as unchanged drug (Fe), and percentage fraction of cumulative fraction of dose recovered in urine as unchanged drug (Fe%). PK parameters were determined by noncompartmental analysis using Phoenix WinNonlin 8.1.

For the SAD phase, blood samples were obtained predose and at 30, 60, 75, 90, 105, 120, and 150 min and at 3, 5, 8, 12, and 24 h following the start of infusion. Urine was collected for PK assessment predose and over the intervals of 0 to 4 h, 4 to 8 h, 8 to 12 h, and 12 to 24 h after the start of infusion.

In the MAD phase, plasma samples for PK analysis were collected (i) on day 1—pre-dose and at 30, 60, 75, 90, 105, 120, and 150 min and at 3, 5, and 8 h following start of the first infusion; (ii) prior to the morning dose (within 10 min) on days 2, 3, 5, 7, 9, 11, and 13; and (iii) predose and 30, 75, 90, 105, 120, and 150 min and 3, 5, 8, and 12 h (day 14), 24, 36 (day 15), and 48 h (day 16) following start of the last infusion. Urine was collected for PK assessment on day 1 predose, over the intervals 0 to 4 h and 4 to 8 h after start of first infusion, and on days 14 and 15 over the intervals 0 to 4 h, 4 to 8 h, 8 to 12 h, and 12 to 24 h after start of day 14 infusion. Total 24-h urine for calculating CrCl was collected from the morning of day −1 to predose on day 1 and on days 15 and 16 over the interval from 24 to 48 h after the start of the day 14 infusion. Urine creatinine concentrations were measured using 24-h collections prior to the first dose on day 1 and over the 24 to 48 h following start of infusion of the last dose (day 14). Plasma and urine samples for SPR206 levels were analyzed using a validated liquid chromatography-tandem mass spectrometry (LC-MS/MS) method. The assay range for SPR206 was 50 to 5,000 ng/ml in plasma and 100 to 50,000 ng/ml in urine. In plasma, assay precision was 1.3 to 7.7%, accuracy was 1.2 to 9.5%, stability was 194 h at 4°C, and reproducibility was 98.4%. In urine, assay precision was 1.7 to 7.5%, accuracy was −4.8 to 7.3%, stability was 147 h at 4°C, and reproducibility was 97.8%.

### Statistical analysis.

Plasma and urine concentrations and PK parameters for SPR206 were summarized for each treatment using descriptive statistics. In the SAD/MAD study, geometric means were calculated for AUC and *C*_max_. Analyses using linear models were performed to assess dose proportionality (both single dose and multiple dose), time dependence, accumulation, and attainment of steady state after multiple doses. Dose-linearity of *C*_max_ and AUC across the dose range was assessed by fitting the power model and testing for β = 1 using a generalized linear model. Point estimates and 90% confidence intervals (CI) using the residual mean square error obtained from the analysis of variance (ANOVA) were constructed for the comparisons between treatments. The point and CI estimates were back-transformed to give estimates of the ratios (%) of the geometric least square means (LSmeans) and corresponding 90% CI.
